# Influence of Bioactive Nutrients on the Atherosclerotic Process: A Review

**DOI:** 10.3390/nu10111630

**Published:** 2018-11-02

**Authors:** Rosa Casas, Ramon Estruch, Emilio Sacanella

**Affiliations:** 1 Department of Internal Medicine, Hospital Clinic, Institut d’Investigació Biomèdica August Pi i Sunyer (IDIBAPS), University of Barcelona, Villarroel, 170, 08036 Barcelona, Spain; rcasas1@clinic.cat (R.C.); restruch@clinic.cat (R.E.); 2 CIBER 06/03: Fisiopatología de la Obesidad y la Nutrición, Instituto de Salud Carlos III, 28029 Madrid, Spain

**Keywords:** CVD, atherosclerosis, hydroxytyrosol, inflammation, nutrients, polyphenols, PUFA, bioactive compounds, phytosterols

## Abstract

The protective effects of a dietary intervention as a useful tool in the prevention of atherosclerosis disease has gained greater attention in recent years. Several epidemiological studies have demonstrated the importance of diet in reducing expensive treatments or possible undesirable side effects. The main aim of this review is to examine the effects of specific nutrients on the development and progression of atherosclerosis in patients with cardiovascular disease. Various mechanisms have been proposed to explain the cardioprotective effect of different nutrients. In this sense, results have shown stabilization of vulnerable atherosclerotic plaques or downregulation of biomarkers related to inflammation through nutrients such as Omega-3 polyunsaturated fatty acids, hydroxytyrosol of extra virgin olive oil, lycopen, phytosterols of plants, or flavonols of fruits and vegetables, among others. The accumulated evidence on the anti-inflammatory effects related to these nutrients is summarized in the present review.

## 1. Introduction

Atherosclerosis is characterized by a multifactorial low-chronic inflammatory process in the arterial wall. Endothelial dysfunction is the first step of this process, which involves some important events including: upregulation of adhesion molecules (E-selectin, vascular cell adhesion molecule-1 (VCAM-1), intercellular cell adhesion molecule-1 (ICAM-1), etc.) leading to recruitment and attachment of circulating monocytes which progressively accumulate low density lipoprotein (LDL) particles and are later oxidized. This is followed by transmigration of monocytes and their conversion to macrophages [1,2]. A large number of chemotactic and growth factors are secreted in this pro-inflammatory scenario in addition to promoting the proliferation of vascular smooth muscle cells (VSMCs) and the progression of atheroma plaque [1,2].

Despite the steady decrease in the global burden of cardiovascular disease (CVD) (28.8%) during the last 10 years, this disease remains the leading cause of death in developed countries (>17.3 million deaths per year in 2013 or 31% of all global deaths) [3,4]. Nonetheless, this trend has slowed due to the presence of risk factors—such as obesity, hyperlipidemia, and diabetes mellitus—which have progressively developed in the global population since the 1980s [5]. It is estimated that one out of every three American adults, that is 85.6 million individuals, has ≥1 type of CVD (concretely, 43.7 million are ≥60 years of age) [4]. Therefore, the leading cause of death attributable to CVD in the United States is coronary heart disease (CHD) with 43.8%, followed by stroke (16.8%), high blood pressure (BP) (9.4%), heart failure (9.0%), diseases of the arteries (3.1%), and other CVDs (17.9%) [3]. In Europe, each year CVD causes 3.9 million deaths and in the European Union, this number is increased by 1.8 million deaths [6]. Indeed, by 2035 these rates are expected to reach 130 million among American deaths by some form of CVD (45.1%) and the total costs of CVD are expected to reach $1.1 trillion [3]. The National Institute of Health estimated that the annual cost of CVD and stroke was $329.7 billion from 2013 to 2014 in the United States [3], being €210 billion a year in the European Union [5]. According to the World Health Organization, 61% of cardiovascular deaths in the world can be explained by eight risk factors—mainly alcohol and tobacco use, high BP, high body mass index (BMI), high blood cholesterol and glucose, low fruit and vegetable intake, and physical inactivity. A reduction of these risk factors can increase the global life expectancy by five years [6,7]. Indeed, according to the US Centers for Disease Control and Prevention, 25% of all deaths by CVD yearly are avoidable. Diet and physical activity are related to seven of the eight risk factors. Accordingly, there is increasing scientific evidence that changes in diet and life habits can prevent the development (primary prevention) or progression (secondary prevention) of CVD, reducing cardiovascular morbidity and mortality [8,9,10,11].

**Figure 1:**
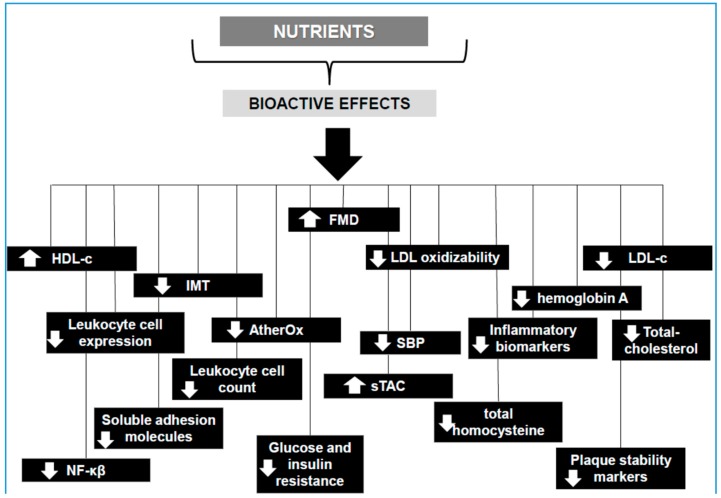
Summary of the main health effects of the nutrients described and their potential effects on the prevention of atherosclerosis. Abbreviations: AtherOx: oxidized low-density lipoprotein (LDL)-β2 glycoprotein1 complex; FMD: flow-mediated dilatation; HDL-c: high-density lipoprotein cholesterol; IMT: intima-media thickness; LDL-c: low-density lipoprotein-cholesterol; NF-κβ: nuclear factor κβ; SBP: systolic blood pressure; sTAC: serum total antioxidant capacity.

With respect to diet, it seems to play a key role in the prevention of atherosclerosis. In fact, nutrition is likely to exert its cardioprotective effects in early stages of atherosclerosis development [12]. In recent years, dietary guidelines are more aimed at promoting the consumption of specific nutrients of natural food sources to obtain better cardiovascular health than simple dietary advice based on the reduction of the consumption of salt, saturated fats, or refined sugar [13]. In fact, there is vast scientific information (clinical data, and epidemiological and experimental studies) suggesting a positive effect between the consumption of specific foods, nutrients and bioactive compounds (mainly antioxidants) and cardiovascular health. Furthermore, low intake or circulatory levels of specific micronutrients—such as magnesium (Mg), phosphorus (P), and calcium (Ca) as well as vitamins A, D or E and the vitamin B group, among others—may also be associated with a greater prevalence of atherosclerosis [14,15,16]. Figure 1 summarizes the main health effects of the bioactive nutrients described throughout this review and their potential effects on the prevention of atherosclerosis.

The aim of this review therefore is to understand how the possible molecular mechanisms are used by dietary components and how a specific nutrient or bioactive compound is involved in the prevention of atherosclerotic disease. In particular, we have included information about the foods, nutrients, or bioactive compounds which are most frequently reported in the scientific literature and have shown a greater effect on the prevention and treatment of atherosclerosis.

## 2. Nutrients in Atherosclerotic Disease

It is important to focus on the possible benefits of the intake of specific nutrients to avoid possible deficiencies of these nutrients which can lead to the development of atherosclerotic disease. We have only included information about fiber, some vitamins, and minerals but no other nutrients—such as carbohydrates, fats. or proteins—which have also been demonstrated to have a certain effect on the risk of developing atherosclerosis.

### 2.1. Fiber

A large number of studies have shown an inverse correlation between diets with a high fiber content and CVD risk [17,18,19]. In addition, a pooled analysis of 18 cohort studies investigated dietary fiber intake and any potential dose–response association with CHD (including 672,408 individuals) and found that higher fiber intake had a significant inverse link with CHD risk in both incidence (7% reduction) and mortality (17% reduction). In addition, the dose–response analysis showed that for each 10 g/day increment in dietary fiber, there is an 8% reduction of all coronary events and a 24% reduction in the risk of death [20]. The authors [20] suggested that the protective factor against CHD was the fiber content of fruit, while vegetable fiber did not show this protection. Fruit fiber decreased CHD risk by 8% (relative risk (RR) = 0.92 95% confidence interval (CI) (0.89–1.01), *p* = 0.01). Other meta-analyses have also described similar results. Threapleton et al. [21] reported a 9% reduction in the risk of CHD (RR = 0.81 95%CI (0.68-0.94)) and CVD (RR = 0.81 95%CI (0.88-0.94)) for each 7 g/day increment in dietary fiber. Another meta-analysis that included 23 studies (involving 937,665 participants and 18,047 patients with CHD) [22] reported a reduction of 16% (RR = 0.84 95%CI (0.82–0.91)) for the intake of fruit per day or 13% (RR = 0.87 95%CI (0.81–0.93)) for the intake of vegetables. Moreover, the dose–response analysis showed reductions of 16% and 18% in CHD after 300 g/day of fruit or 400 g/day of vegetable consumption.

Nonetheless, the number of clinical trials that have shown that fiber intake exerts a protective effect on the progression of atherosclerotic disease still remains limited (Table 1). Chiavaroli et al. [23] carried out a cross-sectional analysis with baseline data from three randomized controlled trials (RCTs). The authors found a significant inverse association between carotid intima media thickness (CIMT) and legume intake, available carbohydrates, glycemic load, and starch but not with dietary fiber after analyzing 325 participants with type 2 diabetes [23]. On the other hand, the results of the PREDIMED (prevention with Mediterranean diet) study [24] pointed out that high fiber intake was inversely associated with carotid atherosclerosis on multivariate analyses (*p* < 0.03). The insulin resistance atherosclerosis study [25] also showed a strong inverse association between whole-grain intake and intima media thickness (IMT) (*p* = 0.005) which even remained significant after adjustment for lipids, adiposity, insulin resistance, nutrient constituents, and a principal components-derived healthy dietary pattern. The cardioprotective effect of fiber on atherosclerotic disease could be explained by the reduction of total serum and low-density lipoprotein cholesterol (LDL-c) concentrations of 9.3 to 14.7 mg/dL and 10.8 to 13.5 mg/dL, respectively [26]. No changes in high-density lipoprotein cholesterol (HDL-c) or triglyceride (TG) concentrations were observed. Additionally, Zhou et al. [27] reported a dose–response answer between increased dietary fiber intake and increased HDL-c. Thus, when the average dietary fiber intake was higher than 30 g/day, HDL-c increased by 10.1%. Other mechanisms attributed to fiber intake and cardiovascular protection might be attributed to a reduction of BP [24,28], improved insulin sensitivity [29], or the prevention of weight gain [24,29].

### 2.2. Micronutrients

Nowadays, there is a vast amount of experimental, epidemiological, and clinical evidence suggesting that micronutrient intake, specifically micronutrients with antioxidant properties, may lower the risk of CVD. Low common carotid arteries (CCA)-IMT may be associated with a high intake and/or circulatory levels of vitamin D and the vitamin B group or Mg [30]. However, this association has not been observed between vitamin E and C and CCA-IMT. Although several meta-analyses reported a possible association between vitamin E and the incidence of CVD [31], many others based on interventional studies found that vitamin E supplementation failed to be atheroprotective in clinical trials in humans [32]. Similar results were shown in a RCT, in which neither supplementation with 400 IU of vitamin E every other day nor 500 mg of vitamin C daily reduced the risk of major cardiovascular events [33]. To the contrary, a recent meta-analysis of 44 RCTs with vitamin C supplementation revealed an improvement in endothelial function in patients with atherosclerosis [34]. On the other hand, a recent systematic review analyzed the link between CIMT and vitamin E (doses varied from 400 to 1200 IU/day), vitamin C (≥250 mg/day) or a combined supplementation of vitamin C and E [35]. This review included 11,307 individuals from the cross-sectional study and 2383 participants with a mean follow-up of 3.1 years. The results did not find any significant association between vitamin C and E supplementation and CCA-IMT. However, a combination of both vitamins might be more effective than their individual effects on the progression of CCA-IMT.

With regard to vitamin D, several epidemiological studies have shown that vitamin D exerts protective effects against atherosclerotic disease through different mechanisms such as modulating immune system response, protecting against endothelial dysfunction, and avoiding VSMC proliferation or migration [36]. Some studies with vitamin D supplementation have described a possible association between supplementation and microvascular calcification [37] contrary to other studies which did not find any significant effect [38]. A cross-sectional study of 107,811 patients reported that patients with optimal vitamin D levels (≥30 ng/mL) had a lower mean total cholesterol (−1.9 mg/dL), lower LDL-c (−5.2 mg/dL), higher HDL-c (4.8 mg/dL), and lower TG concentrations (−7.5 mg/dL) compared with vitamin D deficient patients (<20 ng/mL) [39].

In relation to vitamin E, several human clinical trials have shown a significant decrease in plasma C-reactive protein (CRP), a marker of cardiovascular risk, suggesting that vitamin E and other nutrients also studied, such as fish oil, oleic acid, folic acid, and vitamin B, could reduce cardiovascular risk factors [40] contrary to other studies in which CRP was reduced after carotenoid (vitamin A) and vitamin C consumption but not vitamin E [41]. Finally, De Oliveira et al. [42] examined the associations of dietary micronutrients (heme iron, nonheme iron, zinc (Zn), Mg, β-carotene, vitamin C, and vitamin E) with markers of inflammation and subclinical atherosclerosis (CRP, IL-6, total homocysteine (tHcy), fibrinogen, coronary artery calcium, and common and internal carotid artery IMT). Nonetheless, the authors did not find a strong association between micronutrients and markers of inflammation and subclinical atherosclerosis. On one hand, they found that dietary nonheme iron and Mg intake was inversely associated with tHcy concentrations, while dietary Zn and heme iron were positively associated with CRP levels. On the other hand, no association was found between the dietary intake of β-carotene or vitamin E and markers of inflammation. In addition, vitamin C was positively associated with tHcy concentrations while only Mg showed an inverse association with CCA-IMT.

## 3. Bioactive Compounds and Atherosclerosis

It is known that atherosclerosis burden can be reduced through bioactive compounds such as omega-3 fatty acids, lycopene, or polyphenols, which are natural molecules with great potential to reduce inflammation, LDL-c, and oxidative stress. It should be noted that these molecules can easily be incorporated into the daily diet. In this review, we analyze the bioactive compounds most frequently reported and their effects on atherosclerosis in different epidemiological studies.

### 3.1. Omega-3 Fatty Acids

There is substantial evidence regarding the efficacy of polyunsaturated fatty acids (PUFAs)—such as *n*-3 fatty acid (*n*-3 PUFA), α-linolenic acid (ALA), eicosapentaenoic acid (EPA), and docosahexaenoic acid (DHA)—as potential anti-atherogenic agents of atherosclerotic disease [43]. Some of the mechanisms implicated may be related to the stabilization of vulnerable atherosclerotic plaque, reduced platelet aggregation, or TG levels as well as anti-inflammatory effects. [44,45]. Thus, marine *n*-3 PUFA may lead to decreased infiltration of inflammatory and immune cells such as monocytes or lymphocytes into the plaque and/or immunomodulation of these cells, thereby decreasing their proinflammatory activity [46]. In an interventional study in patients awaiting carotid endarterectomy, Thies et al. [47] showed that administering marine *n*-3 PUFA (1.4 g EPA + DHA/day) as dietary fish oil supplements in patients with advanced atherosclerotic plaque was associated with increased plaque stability, and less inflammation and infiltration of macrophages and lymphocytes. Similar results were found in the Omacor Carotid EnArterectomy iNtervention (OCEAN) study, which found mRNA levels for matrix metalloproteinases (MMP) MMP-7, MMP-9, and MMP-12 to be lower in plaque from patients who had received marine n-3 PUFA with the administration of 1.8 g EPA + DHA/day [48]. Indeed, MMPs play a significant role in weakening the fibrous cap and promoting plaque vulnerability [2]. Some cross-sectional studies have shown that n-3 PUFA are inversely associated with CCA-IMT, a marker of atherosclerosis and a predictor of cardiovascular risk [49]. For example, He et al. [50] analyzed 5480 adults aged 45–84 years who were free of CVD and found that the dietary intake of long-chain n-3 PUFAs (0.69 (95% CI: 0.55, 0.86; *p* < 0.01)) or non-fried fish (0.80 (95% CI: 0.64, 1.01; *p* = 0.054)) was inversely associated with CCA-IMT as a marker of subclinical atherosclerosis, with the results being dependent on the type of fish consumed. In addition, an observational case series study of a cohort of 600 men with CVD who received fish oil supplementation showed reduced markers of atherothrombotic risk [51]. Moreover, a retrospective study in 160 Japanese volunteers with CVD reported that low DHA levels were correlated with reduced endothelial function, measured as flow-mediated dilation (FMD) [52]. This observation was similar to an earlier study that reported an improvement of endothelial function and arterial stiffness with a parallel anti-inflammatory effect after treatment with n-3 PUFAs in patients with metabolic syndrome (MetS) [53]. However, a recent study in patients with type 2 diabetes mellitus (T2DM) and established atherosclerotic CVD did not find an improvement in the endothelial function indices (FMD and nitroglycerin-mediated dilation) after three months of high-dose n-3 PUFA treatment [54]. A meta-analysis of 38 clinical intervention studies reported that increased consumption of EPA and DHA through either supplementation or consumption of enriched foods was associated with a 20–30% reduction in serum TG levels (≥4 g/day) in healthy patients and in patients with borderline hyperlipidemia [55]. More recently, Hidayat et al. [56] observed a reduction in heart rate with n-3 long-chain PUFA supplementation. However, when DHA or EPA was administered alone, the heart rate was slowed by DHA but not EPA.

### 3.2. Lycopene

Lycopene is a carotenoid present in tomatoes and tomato products, grapefruit, watermelon, and papaya. Several studies have reported that lycopene may protect against the development of atherosclerosis because of its antioxidant effects [57]. In this sense, some authors have suggested that high carotenoid levels in serum may slow early stages of atherosclerosis progression [58]. Xu et al. [58] found serum lutein to be inversely associated with IL-6 (*p* < 0.001) and zeaxanthin with VCAM-1 (*p* = 0.001) and apoE (*p* = 0.022), and finally, lycopene was inversely associated with VCAM-1(*p* = 0.011) and LDL-c (*p* = 0.046). Karppi et al. [59] suggested that lycopene or processed tomato intake may reduce CCA-IMT in vessel walls after examining the effect of carotenoids on the early development of atherosclerosis. However, Sesso et al. [60] did not find any correlation between the risk of CVD and higher plasma lycopene concentrations after examining 499 cases of CVD and an equal number of older men (69.7 ± 8.1 years) free of CVD over a follow-up period of 2.1 years. These same authors [61] conducted a study in 28,345 women free of CVD and cancer over a mean follow-up of 4.8 years and found higher plasma lycopene concentrations to be associated with a lower risk of CVD in women. Interventional studies analyzing the possible cardioprotective effect of raw tomatoes and tomato sauce intake on proinflammatory biomarkers and leukocyte molecules related to the early development of atherosclerosis also concluded that tomato intake has beneficial effects on cardiovascular risk factors, especially cooked tomatoes enriched with oil [62]. However, on assessing whether the consumption of tomato-based foods affects recognized biomarkers of CVD risk in 225 healthy middle-aged participants, Thies et al. [63] did not find any changes in inflammatory markers, markers of insulin resistance and sensitivity, lipid concentrations or arterial stiffness after dietary intervention with a control diet (low in tomato-based foods), a high-tomato-based diet, or a control diet supplemented with lycopene capsules (10 mg/day) for 12 weeks. In addition, several studies have reported an improvement of oxidized-LDL (oxLDL) cholesterol levels after lycopene intake, suggesting the possible role of lycopene in the prevention of oxidative stress-related diseases [30,64]. Abete et al. [64] evaluated the effects of the consumption of 160 g of two tomato sauces with different concentrations of lycopene on oxidative stress markers and found that regular consumption of the high-lycopene tomato sauce induced a significant reduction in oxLDL cholesterol levels (−9.27 ± 16.8%; *p* < 0.05), demonstrating the preventive effects of lycopene against oxidative stress-related diseases.

### 3.3. Plant Sterols and Stanols

Phytosterols (plant sterols) are natural sterols of plant origin, the most abundant being sitosterol [57]. The main natural sources of phytosterols are vegetable oils [57]. Sitosterol has a structure similar to that of cholesterol, but this sterol contains an extra ethyl group at position C-24, lowering cholesterol absorption in the intestine (by 5% to 15% in a dose-dependent manner), and effectively and safely downregulating plasma cholesterol levels [65,66].

There is currently a great deal of scientific evidence demonstrating that a daily dose of 2–3 g of phytosterols leads to a significant reduction of 6–15% in LDL-c [67,68,69]. The cardioprotective mechanism of phytosterols is the competition of sterols with cholesterol in the lumen of the intestine after dietary and biliary cholesterol uptake [69,70]. In addition, a meta-analysis [71] reported significant reductions in LDL-c concentrations of 8.8% after the intake of 2.15 g/day of phytosterols. Moreover, a systematic review and meta-analysis of 20 eligible RCTs, including a total of 1308 subjects reported LDL-c concentrations (−14.3 mg/dL) to be significantly reduced, but no changes in CRP levels were observed (*p*-value = 0.073) [72]. In 2009, Scholle et al. [73] published a meta-analysis evaluating 8 RCTs (306 hypercholesterolemic patients). These authors found that the use of statins combined with plant sterols or stanols decreased total cholesterol (−14.01 mg/dL, *p* < 0.0001) and LDL-c (−13.26 mg/dL, *p* < 0.0001). However, no changes in HDL-c or TG were observed.

Several clinical data in humans support the role of phytosterols in decreasing LDL-c levels. Andersson et al. [70] reported that a diet with a high level of plant sterols (mean intake of 463 mg daily) in 22,256 study participants was inversely correlated with lower plasma LDL-c levels. This finding is consistent with another epidemiological study in which a significant reduction in LDL-c levels (0.26 mmol/L) was described after 233 subjects received supplementation with phytosterols during 12 weeks [74]. In a retrospective cohort study in 3829 participants (43 of whom were receiving treatment with statins) [75], a daily intake of ≥20 g of phytosterols (in the form of sterol- or stanol-enriched margarine) was associated with a significant decrease in cholesterol levels (−0.32 mmol/L). This increase was dose-dependent with a decrease of −0.0094 mmol/L for each gram of enriched margarine. This cholesterol-lowering was also observed in statin plus pytosterol users. Escurriol et al. [76] evaluated if increased plasma phytosterols lead to CHD and found that CHD risk and plasma phytosterol intake was not related to the apolipoprotein E (APOE) genotype.

## 4. Polyphenols

The progression of atherosclerosis can be attenuated by the effect of polyphenols. Among other effects, polyphenols can prevent leukocyte migration, reduce production of adhesion molecules, improve coagulation activity and endothelial function, reduce BP due to their antioxidant capacity, modulate the transcription of nuclear factor-κβ (NF-κβ), or inhibit the encoding of pro-inflammatory cytokines [57]. Polyphenols also have the capacity to generate nitric oxide, which acts as a potent vasodilator on the endothelial surface and in the early stages of atherosclerosis, improve antioxidant status, and decrease inflammatory cytokine levels (tumor necrosis factor-alpha (TNF-α), interleukin (IL), MMPs) and adhesion molecules (VCAM-1, ICAM-1, and selectins) [77].

### 4.1. Flavonoids

Epidemiological and experimental evidence suggest that there is a protective relationship between the consumption of foods rich in flavonoids (such as tea, berries, cocoa, chocolate, and wine) and the risk of CVD [78]. Concretely, flavonoids may act on the growth of atherosclerotic plaque decreasing adhesion molecule expression and inflammation and reducing the capacity of macrophages to oxidate LDL-c [79]. Some of main foods studied are:

#### 4.1.1. Flavanols

In relation to flavonoid-rich foods such as cocoa, which is rich in flavanols, epicatechin, catechin, and proanthocyanidins, several reviews and meta-analyses have examined the association of cacao consumption with different cardiovascular risk factors such as BP, endothelial function, blood lipids, and platelet function and cardiovascular health [80,81,82,83]. On one hand, the daily intake of 50 g of dark chocolate improves endothelial function, measured as FMD, by 3.99% in acute and 1.45% in chronic intake [80]. In addition, a meta-analysis of 10 RCTs reported a decrease of 4.5 mmHg in systolic BP (SBP) and 2.5 mmHg in diastolic BP (DBP) with the intake of flavanol-rich cocoa products [81]. Ostertag et al. [82] concluded that 100 mg of flavanols led to a 3–11% inhibition of platelet function, suggesting that the consumption of 100 g of dark chocolate with 70% cocoa solids could result in an effect similar to that of 81 mg of aspirin in an acute setting. Finally, Jia et al. [83] showed that cocoa consumption significantly lowered LDL-c by 5.87 mg/dL (95% CI: −11.13, −0.61; *p* < 0.05) in the short-term. However, the authors did not find any evidence of a dose–effect relationship, any effect in healthy subjects, or any change in HDL-c levels.

Several RCTs have also shown a relationship between cocoa polyphenols and atherosclerosis-related inflammatory markers (Table 1). In this regard, Monagas et al. [84] suggested that chronic consumption of cocoa powder may modulate the expression of adhesion molecules on leukocyte surfaces as well as soluble adhesion molecules (sP-selectin and sICAM-1) concentrations, all of which are related to early stages of atherosclerosis in subjects at high risk of CHD. Another crossover study [85] in 18 healthy subjects evaluated the effect of acute cocoa consumption on different matrices (water and milk) related to the bioavailability of cocoa polyphenols in NF-κβ activation and the expression of adhesion molecules. NF-κβ drives the expression of proinflammatory genes and initiates the production of signals that initiate adaptive immunity [2]. The authors suggested that cocoa consumption could inhibit the NF-κβ-dependent transcription pathway and the interaction with certain cytokines. Moreover, this effect may be modulated by the food matrix. Esser et al. [86] carried out another crossover study to investigate if flavanol-enriched chocolate consumption increased endothelium-dependent vasodilation. These authors were not only interested in endothelial health, but also investigated whether regular consumption of dark chocolate also affects other markers of endothelial health, and whether chocolate enrichment with flavanols has additional benefits. They found that in addition to improving vascular function, chocolate intake also lowers the adherence capacity of leukocytes in the circulation.

#### 4.1.2. Catechins

On the other hand, green tea is a rich source of flavonoids, especially catechins (90%) such as epicatechin, epicatechin gallate, epigallocatechin, and epigallocatechin gallate. The greatest fraction of black tea polyphenols is composed of thearubigins and total flavonoids (47%) [87]. A great number of reviews and meta-analyses have reported the cardioprotective effects of tea consumption. In a review of 21 prospective studies, Gardner et al. [88] found that daily consumption of three or more cups of black tea was associated with a reduction in CHD risk. Grassi et al. [87] also concluded that there was an inverse association between black and green tea consumption and CVD after the review of 15 epidemiological studies. These results are in accordance with a meta-analysis that included nine prospective studies (4378 strokes among 194,965 individuals) which concluded that drinking ≥3 cups daily of tea (black or green tea) reduces the risk of stroke by 21% compared to the consumption of <1 cup per day [89]. Several meta-analyses of RCTs have also studied the benefits of tea in CVD. On one hand, Hooper et al. [80] reported that acute black tea intake increased SBP (5.69 mm Hg) and DBP (2.56 mm Hg) and also increased FMD (3.40%). On the other hand, green tea reduced LDL-c (−0.23 mmol/L) concentrations, but there was no significant effect on HDL-c.

As shown in Table 1, a crossover study of 21 healthy postmenopausal women [90] showed that the intake of green and black tea led to a significant increase in FMD (*p* < 0.001; both). In another crossover study involving five different treatments (0, 100, 200, 400, and 800 mg tea flavonoids/day), Grassi et al. [91] reported that black tea consumption dose-dependently improved endothelial function in healthy men. Even 100 mg/d (<1 cup of tea) increased FMD compared with controls (*p* = 0.0113). In addition, black tea intake decreased SBP (−2.6 mmHg, *p* = 0.0007) and DBP (−2.2 mmHg, *p* = 0.006) as well as the stiffness index (*p* = 0.0159). Suzuki-Sugihara et al. [92] suggested that green tea catechins may be rapidly incorporated into LDL particles and play a key role in reducing LDL oxidation in humans. Their results suggest that taking green tea catechins could be effective in reducing the risk of atherosclerosis associated with oxidative stress.

#### 4.1.3. Quercetin

Fruits and vegetables like apples, onions, cherries, and grapes have a high quercetin content, one of the most important flavonoids in the human diet [93]. The atheroprotective effects of quercetin can be attributed to its antioxidant and anti-inflammatory effects as well as its positive effects on diabetes or obesity [93]. Dower et al. [94] investigated the role of quercetin in atherosclerosis in 37 healthy (pre)hypertensive men and women. The participants took 100 mg of (-)-epicatechin, 160 mg of quercetin-3-glucoside or placebo capsules daily for four weeks, in random order. The main finding was that quercetin may improve endothelial function and reduce inflammation, two main determinants of atherosclerosis. Larson et al. [95] also carried out a randomized, double-blind, placebo-controlled, crossover trial to study if quercetin intake reduced BP in hypertensive individuals. The authors observed a decrease of BP in stage 1 hypertensive men after the administration of a single dose of purified quercetin aglycone, while BP remained unchanged in normotensive volunteers. Moreover, angiotensin-converting enzyme concentrations, endothelin-1 levels, nitrites, and brachial artery FMD were not affected by quercetin.

#### 4.1.4. Anthocyanins

These compounds are mostly found in blueberries, cranberries, bilberries, chokeberries, and elderberries [96]. Several meta-analyses of RCTs [97,98] have studied the effects of berry (anthocyanins) consumption on CVD risk factors, reporting significantly lower LDL-c, SBP, fasting glucose, BMI, hemoglobin A1c or tumor necrosis factor-α (TNF-α) levels and increased HDL levels. Kuntz et al. [99] reported that anthocyanins can exert their benefits on cardiovascular health through not only their antioxidant effects but also by downregulation of pro-inflammatory parameters. In this randomized study, these, authors reported that the ingestion of anthocyanin-rich beverages significantly increased plasma superoxide dismutase (SOD) and catalase activities (*p* < 0.001; both) as well as Trolox equivalent antioxidant capacity (*p* ≤ 0.01), while concentrations of malondialdehyde, a marker of lipid peroxidation, were significantly decreased (*p* < 0.001). In another interventional study [100] including 42 volunteers who were overweight and smokers, significant reductions in oxLDL and 8-iso-prostaglandin F2α, proposed as a new indicator of oxidative stress, were observed after 40 days of supplementation with a standardized extract of maqui berry (162 mg anthocyanins), suggesting that a polyphenol-rich diet may exert health-promoting effects by reducing oxidative stress. A recent RCT [101] also examined the effects of long-term supplementation with purified anthocyanins (320 mg anthocyanin/capsule) on platelet chemokines, which are chemotactic cytokines and can be induced during immune response to recruit cells from the immune system [2]. After 24 weeks, the intervention group showed significant reductions of different chemokines (CXCL7 (−9.95%), CXCL8 (−6.07%), CXCL12 (−8.11%), and CCL2 (−11.63%)) levels compared with the placebo group. These results suggest that platelet chemokines may be targets of anthocyanins in the prevention of atherosclerosis. Anthocyanins were also administered at a dose of 320 mg/day to hypercholesterolemic patients for a period of 24 weeks in a randomized, double-blind, placebo-controlled trial [102]. Anthocyanin intake significantly decreased plasma concentrations of β-thromboglobulin, P-selectin, and RANTES (regulated on activation, normal T cell expressed and secreted. Later, in vitro experiments in patients receiving anthocyanins showed a significant reduction in the secretion of pro-inflammatory and pro-thrombotic factors.

#### 4.1.5. Isoflavones

Soy and soy products also contain significant amounts of isoflavones and their atheroprotective effects can be attributed to the associated reduction in cholesterol levels and a reduction in oxidative stress in human studies [103]. A meta-analysis of 133 RCTs carried out by Hooper et al. [80] found a significant reduction in DBP (−1.99 mmHg) and LDL-c (−0.19 mmol/L) after chronic soy protein isolate consumption (but not whole soy or soy extracts). The mechanism by which soy protein intake leads to significant reductions of LDL-c may be due to high isoflavone intake [104,105]. Moreover, to achieve a clinically important reduction in LDL-c, the consumption of 20–56 g soy protein isolate powder daily (up to 75% of the usual protein intake) is required.

No significant effects on HDL-c, SBP, or FMD were observed after the consumption of a protein isolate or isoflavone extracts. Tokede et al. [106] also studied the relation between soy protein consumption and serum lipid concentrations in humans in a meta-analysis of 35 RCTs and found a significant decrease in LDL-c levels of −4.33 mg/dL, TG-4.92 m/dL and total cholesterol −5.33 mg/dL. There was also a significant increase in serum HDL-c concentrations of 1.40 mg/dL. On the other hand, in a RCT [107] including postmenopausal American women receiving soy protein supplementation daily (25 g/day), subclinical atherosclerosis progression lowered by 16% compared to the placebo group, although this treatment effect was not statistically significant. In addition, in women who were randomized within five years of menopause, soy protein intake reduced carotid thickness progression by a mean of 68%. In a cross-sectional study of 2135 women and 804 men aged 50–75 years [108], women showed a lower presence of elevated total cholesterol, dyslipidemia, hyperuricemia, and a lower number of cardiometabolic disturbance components when soy consumption was higher.

### 4.2. Stilbens

Resveratrol is a polyphenol found in the skin of grapes, berries, and peanuts [109]. Resveratrol possesses antioxidant properties [110], increases the production of nitric oxide synthase (NOS) [111] and improves mitochondrial function [112]. A large number of epidemiological studies have reported the beneficial effects of resveratrol on hypertension, atherosclerosis and ischemic heart diseases [113]. In a meta-analysis of six RCTs evaluating the effects of resveratrol on SBP and DBP, Liu et al. [114] showed that higher resveratrol consumption (≥150 mg/day) significantly reduced SBP by −11.90 mmHg (*p* = 0.01), whereas a lower dose of resveratrol did not show a significant lowering effect on SBP. These results are in accordance with a recent meta-analysis of 17 RCTS and suggests that the use of resveratrol promotes cardiovascular health, mainly at a high daily dose (300 mg/day) and in diabetic patients [115]. Several clinical trials have also studied the effect of resveratrol on lipids. As shown in Table 1, Bhatt et al. [116] hypothesized whether oral supplementation of resveratrol (250 mg/day) improved glycemic control and the associated risk factors in patients with T2DM. The results revealed that supplementation with resveratrol for three months significantly improved hemoglobin A (1c) concentrations (−0.33 ± 0.04, *p* = 0.02), total cholesterol (−14.32 ± 5.31, *p* = 0.004) and LDL-c (−12.45 ± 6.95, *p* = 0.05) as well as SBP levels (−11.78 ± 0.73, *p* = 0.002) in T2DM. Similar results were described in the study by Movahed et al. [117] in which diabetic patients were supplemented with 1 g/day of resveratrol for 45 days. In another study, Tomé-Carneiro et al. [118] investigated the effects of a six-month intake of grape supplement containing 8 mg resveratrol on oxLDL, apolipoprotein-B (ApoB), and serum lipids in statin-treated patients on primary CVD prevention. There was a reduction in atherogenic markers including oxLDL, ApoB, and LDL-c. A recent study [119] showed that resveratrol may be beneficial in preventing the development of atherosclerosis induced by diabetes to decrease the cardio-ankle vascular index (CAVI) (−0.4 ± 0.7) as a clinical surrogate marker of atherosclerosis, SBP (−5.5 ± 13.0 mmHg) and diacron-reactive oxygen metabolites (d-ROMs) (−25.6 ± 41.8 U.CARR) as a marker of oxidative stress. A randomized, double-blind, placebo-controlled clinical trial [120] also studied the effects of resveratrol on markers of oxidative stress in 48 patients with T2DM (aged 30–70 years). In this case, after supplementation with 800 mg/day of resveratrol for eight weeks, it was found that resveratrol decreased plasma protein carbonyl content and ROS levels in peripheral blood mononuclear cells (PBMCs) and significantly increased plasma total antioxidant capacity and total thiol content. Furthermore, the expression of Nrf2 and *superoxide dismutase* (SOD) was significantly increased after resveratrol consumption. Finally, in another placebo-controlled clinical trial [121] with a four-week supplementation with resveratrol in 44 healthy individuals, the levels of pro-inflammatory cytokine, IL-8 (*p* = 0.022), and interferon gamma (*p* = 0.033) and the expression of cell adhesion molecules such as sICAM-1 (*p* = 0.037) and sVCAM-1 (*p* = 0.017) significantly decreased. However, no significant change was observed in other inflammatory markers such as TNF-α, IL-1β, or IL-6. Fasting insulin levels were also reduced in the resveratrol group (*p* = 0.045).

### 4.3. Other Polyphenols

Several clinical, epidemiological, and experimental studies suggest that the consumption of olive oil—and more specifically, extra virgin olive oil (EVOO)—reduces the incidence of certain diseases such as hypercholesterolemia, atherosclerosis, hypertension, thrombotic risk, oxidation and oxidative stress, obesity, and the MetS [122]. Hydroxytyrosol (HT) and oleuropein and tyrosol seem to be the most important phenolic fractions responsible for the prevention of the development of atherosclerosis. These phenolic compounds may reduce endothelial dysfunction [123] and decrease the concentration and atherogenicity of LDL-c [124] as well as improve antioxidant effects [125] and HDL-c function [126], and inhibit platelet aggregation [127].

A recent review [122] examined the biological properties and antioxidant capacity of EVOO against diseases such as atherosclerosis, diabetes, obesity, and cancer. Furthermore, it has been reported that HT could modulate the transcription of NF-κβ and inhibit the encoding of cytokines (TNF-α, IL-1, IL-6, or IL-7), chemokines and other proinflammatory biomarkers [128]. Several sub-studies of the PREDIMED trial [129,130,131,132,133,134] also reported that a Mediterranean diet (MD) supplemented with EVOO may downregulate the expression of leukocyte adhesion molecules, soluble endothelial adhesion molecules (sICAM-1, sVCAM-1, sP-Selectin, etc.), several cytokines (IL-6, IL-8, TNF-α), and chemokines (MCP-1, ENA78, RANTES, etc.) as well as molecules related to the vulnerability of atheroma plaque (IL-18, MMP-9, IL-10, TGF-β1, etc.). These results were observed at 3 and 12 months and were maintained or even improved at three and five years. Camargo et al. [135] studied the effects of dietary fat on the expression of genes related to inflammation such as NF-κβ, IL-6, and TNF-α, as well as those related to plaque stability (MMP-9) during the postprandial state in 20 healthy elderly people following three different diets for three weeks each. The results showed that consumption of a MD reduces the postprandial inflammatory response (NF-κβ, MCP-1, MMP-9, and TNF-α expression) compared with two other diets (*p* < 0.05; all). Atherosclerosis and the subsequent development of CHD are triggered by oxLDL, the main key factor of these pathogenic events [43].

Many in vivo human studies have demonstrated that increased consumption of EVOO phenolic compounds (mainly HT) leads to a decrease in oxLDL and an increase in HDL-c levels [136,137,138]. A recent study [139] showed that a MD supplemented with EVOO decreased LDL atherogenicity in a random sub-sample of individuals from the PREDIMED study, which could partially explain the cardioprotective benefits of this dietary pattern. Castañer et al. [140] found that polyphenol-rich EVOO might exert a reduction in LDL oxidation and the gene expression of the CD40 ligand (CD40L), IL-23α subunit p19 (IL23A), adrenergic β-2 receptor (ADRB2), oxLDL (lectin-like) receptor 1 (OLR1), and IL-8 receptor-α (IL8RA), all of which are genes involved in the atherogenic and inflammatory processes in which LDL oxidation is involved. A study carried out by Widmer et al. [141] also found significant reductions in proinflammatory markers and oxidative stress after the consumption of 30 mL of EVOO.

## 5. Conclusions

According to the data presented, there are a great number of nutrients or bioactive compounds that seem to have beneficial effects on CVD. These effects could be explained by the modulation of immune system response, endothelial dysfunction, avoiding VSMC proliferation or migration, reducing oxidative stress or stabilization of vulnerable atherosclerotic plaque, among others. Based on this evidence, diets such as the MD could be potentially efficacious in the primary and secondary prevention of CVD. Therefore, nutritional recommendations should be established, and the most adequate intake of bioactive compounds should be determined in order to reduce the incidence of CVD. To do this, it is necessary to conduct new randomized clinical intervention trials that specifically use and/or combine these nutrients, active compounds, or specific foods with each other in order to resolve questions such as how much, how, and when as well as possible nutrient–drug interactions.

## Figures and Tables

**Table 1:** Possible mechanisms by which nutrient/bioactive compounds intake can exert a protective effect on the progression of atherosclerotic.

Nutrient/Bioactive Compound	Study Design	Participants	Type of Study	Findings
***Fiber*** Chiavaroli et al. [23]	Ultrasonographic carotid intima media thickness (CIMT) at baseline, and 7-day food records	325 participants with type 2 diabetes from three randomized controlled trials collected	Cross-sectional analysis	CIMT was significantly inversely associated with dietary legume intake (*β* = −0.019, *p* = 0.009), available carbohydrate (*β* = −0.004, *p* = 0.008), glycemic load (*β* = −0.001, *p* = 0.007) and starch (*β* = −0.126, *p* = 0.010), and directly associated with total (*β* = 0.004, *p* = 0.028) and saturated fat (*β* = 0.012, *p* = 0.006)
Buil-Cosiales et al. [24]	MD + EVOO (50 mL daily) or nuts (30 g daily) vs. a LFD. Dietary habits were assessed with 137-item FFQ and a 14-item questionnaire. Ultrasonographic CCA-IMT measurement at baseline.	457 men and women aged between 55 and 80 years at high cardiovascular risk.	Cross-sectional study.	Non-adjusted model: significant inverse correlation between fiber intake and IMT (*r* = −0.27, *p* < 0.001) and adjusted-model (*p* < 0.03) for >35 g fiber/day in adults.
Mellen et al. [25]	114-item FFQ. Ultrasonographic CCA-IMT measurement at baseline and at 2 years.	Multiethnic cohort with 1178 participants (56% female) aged 40–69 years with a range of glucose tolerance (normal, impaired, and diabetic).	Multicenter, prospective, observational study.	Whole-grain intake was inversely associated with CCA-IMT (*β* ± SE: −0.043 ± 0.013, *p* = 0.005).
**Micronutrients** Ponda et al. [39]	Participants were stratified according to deficient (<20 ng/mL), insufficient (20–29 ng/mL), and optimal (≥30 ng/mL) vitamin D levels.	107,811 participants. Aged between 40–80 years.	Cross-sectional study.	Optimal vitamin D levels (≥30 ng/mL) were associated with lower mean total cholesterol, LDL-c, and TGs and higher HDL-c (*p* < 0.0001; all).
Carrero et al. [40]	Over one year, intake of 500 mL/day of a fortified dairy product containing EPA, DHA, oleic acid, folic acid, and vitamins A, B-6, D, and E (supplemented group) or 500 mL/day of semi-skimmed milk with added vitamins A and D (control group).	Patients with MI with a mean age of 52.6 ± 1.9 years in the supplemented group and 57.4 ± 1.8 years in the control group.	Longitudinal, randomized, controlled, double-blind intervention study.	↓ Plasma total and LDL-cholesterol, apolipoprotein B, and CRPin the supplemented group (*p* < 0.05). ↓ Plasma tHcy in both groups.
De Oliveira et al. [41]	120-item, self-administered FFQ was used to assess usual food intake over the previous year.	5181 participants from the multi-ethnic study of atherosclerosis. Aged 45–84 years and free of diabetes and CVD.	Cross-sectional study.	Dietary nonheme iron and Mg intakes were inversely associated with tHcy concentrations (*p*-trend < 0.001 for both). Dietary Zn and heme iron were positively associated with CRP (*p*-trend = 0.002 and 0.01, respectively). A positive association was found between tHcy concentrations and Vitamin C (*p*-trend = 0.01) and an inverse association between Mg with CCA-IMT (*p*-trend = 0.001).
**n-3 PUFA** Cawood et al. [48]	Daily intake of placebo or n-3 PUFA (1.8 g EPA + DHA/day) capsules until surgery (median 21 days).	121 patients awaiting carotid endarterectomy. >18 years of age.	Double-blind, placebo-controlled design.	n-3 PUFA group: ↑EPA (*p* < 0.0001) and ↓ foam cells (*p* = 0.0390), mRNA for MMP-7 (*p* = 0.0055), -9 (*p* = 0.0048) and −12 (*p* = 0.0044) and for IL-6 (*p* = 0.0395) and ICAM-1 (*p* = 0.0142).
Franzese et al. [51]	Compared use of fish oil supplementation in various subgroups: non lipid-lowering therapy vs. lipid-lowering therapy.	600 men with CVD, aged 64.4 ± 10.1 year.	Observational case series study.	VLDL, IDLs, remnant lipoproteins, TG, LDL, AtherOx levels, collagen-induced platelet aggregation, thrombin-induced platelet-fibrin clot strength, and shear elasticity (*p* < 0.03 for all).
Tousoulis et al. [53]	Daily intake of n-3 PUFAs (2 g/day) or placebo for 12 weeks. 4-week washout periods.	29 subjects, 14 females, and 15 males with MetSaged 44 ± 12 years.	Double-blind, placebo controlled, cross-over trial.	PUFAs: Significant improvement of FMD and PWV (*p* < 0.001 for all). ↓ IL-6, PAI-1 (*p* = 0.003; both) ↓ TG and total cholesterol levels
Siniarski et al. [54]	Daily intake of n-3 PUFAs (2 g/day) or placebo for 3 months. 4-week washout periods.	74 patients with established ASCVD and T2DM.	Two-center, prospective randomized double-blind, placebo-controlled study.	Did not improve endothelial function indices (FMD and NMD).
**Lycopene** Karppi et al. [59]	Determination of plasma carotenoid concentrations and measurements of CCA-IMT by B-mode ultrasound. ~20 years follow-up.	1212 elderly Finnish men aged 61–80 year.	Prospective study.	Higher concentrations of plasma β-cryptoxanthin (*p* = 0.043), lycopene (*p* = 0.045), and α-carotene (*p* = 0.046) were associated with lower CCA-IMT.
Sesso et al. [61]	Plasma lycopene, other carotenoids, retinol, and total cholesterol were measured. Mean follow-up of 4.8 years.	28,345 female US health professionals free of CVD and cancer. Aged 45 years.	A prospective, nested, case-control study.	Higher plasma lycopene concentrations were associated with a lower risk of CVD in women.
Valderas-Martinez et al. [62]	Intake of 7.0 g of RT/kg BW, 3.5 g of TS/kg BW, 3.5 g of TSOO/Kg BW and 0.25 g of sugar dissolved in water/kg BW on a single occasion on four different days.	40 healthy subjects (mean age of 28 ± 11 years).	Open, prospective, randomized, cross-over, controlled feeding trial.	RT: ↓ SBP, total cholesterol, TGs, or MCP-1 and ↑ folic acid and IL-10. TSOO: ↓ SBP, DBP, total cholesterol, TGs, IL-6, IL-18, MCP-1 and VCAM-1 and ↑ folic acid, IL-10. ↓ LFA-1 from T-lymphocytes and CD36 from monocytes.
Thies et al. [63]	A control diet (low in tomato-based foods), a high-tomato-based diet, or a control diet supplemented with lycopene capsules (10 mg/day) for 12 weeks.	225 healthy volunteers (94 men and 131 women), moderately overweight (BMI:18.5–35) and aged 40–65 years.	Single-blind, randomized controlled dietary intervention study.	No changes in systemic markers (inflammatory markers, markers of insulin resistance, and sensitivity), lipid concentrations or arterial stiffness in all interventions.
Abete et al. [64]	Effect of the consumption of 160 g of two TSs with different concentrations of lycopene on oxidative stress markers: high-lycopene TS (27.2 mg of lycopene) vs. commercial TS (12.3 mg of lycopene). 4 weeks separated by a 2-week washout period.	32 healthy patients (18 males and 14 females). Aged between 18–50 years with a BMI of 18.5–29.9 kg/m^2^.	Double-blind crossover nutritional intervention.	High-lycopene TS: ↓ LDL-ox (−9.27 ± 16.8%; *p* = 0.014).
**Plant Sterols and Stanols** Ras et al. [74]	20 g/day of low-fat spread without (control) PS vs. with added PSs (3 g/day) during 12 weeks. Measurement of: FMD, serum lipids, arterial stiffness, BP.	232 hypercholesterolemic participants (healthy men and postmenopausal women), aged 40–65 years	Double-blind, randomized, placebo-controlled, parallel design.	Lower LDL-c levels (average of 0.26 mmol/L)
Eussen et al. [75]	Questionnaires on health and food intake were used to assess PS intake. Measurement of serum lipids. 5-year follow-up	3,829 men and women (aged 31–71 years).	Retrospective cohort study.	Significant decrease of cholesterol (−0.32 mmol/L) with increasing intake of enriched margarine.
Escurriol et al. [76]	FFQ was used to assess PS intake. Measurement of serum lipids	Healthy men and women: 299 developed CHD and 584 as controls, aged between 30 and 69 years (Spanish EPIC cohort)	Case-control study.	High levels of PS →↑ HDL-c, cholesterol/HDL ratios, and ↓ glucose, TG and lathosterol, (*p* < 0.02; all). No correlation between APOE genotype and CHD risk or plasma phytosterols
***Flavonoids*** Monagas et al. [84]	4-weeks of intervention of: 40 g cocoa powder with 500 mL skim milk/d (C + M) or only 500 mL skim milk/d (M). Daily: 40.41 mg (+)-catechin 46.08 mg (−)-epicatechin 36.54 mg procyanidin B2 495.2 mg tot.PPh 425.7 mg tot.Pr	42 high-risk volunteers (19 men and 23 women). Aged ≥55 years.	Randomized crossover study.	C + M: ↓ VLA-4, CD40, CD36 (monocytes) (*p* ≤ 0.028; all) ↓ P-selectin and ICAM-1 (*p* = 0.007; both) Non-significant changes: ↓ VCAM-1 and MCP-1 No effect: hs-CRP, IL-6, E-selectin
Vázquez-Agell et al. [85]	Acute intervention (6 h) of: 40 g Cocoa powder with 250 mL milk or water (W). Daily: 40.41 mg (+)-catechin 46.08 mg (−)-epicatechin 36.54 mg procyanidin B2 495.2 mg tot.PPh 425.7 mg tot.Pr	18 healthy volunteers: 9 men and 9 women, aged 19–49 years).	Randomized crossover study.	↓ NF-κβ (cacao + W; *p* < 0.05) ↓ E-selectin (cacao + W; *p* = 0.028) ↓ ICAM-1 (cacao + W or M; *p* ≤ 0.026, both) No effect: VCAM-1
Esser et al. [86]	Daily consumption of high flavanol chocolate (HFC) and normal flavanol chocolate (NFC). 4-week intervention.	Healthy overweight men (age 45–70 years).	Randomized crossover study.	HFC intake: ↑ FMD 1% (*p* = 0.010) ↓ ICAM-1, ICAM-3 (*p* ≤ 0.023; both) ↓ Leukocyte cell count (*p* = 0.023) ↓ Leukocyte adhesion marker expression (*p* ≤ 0.047; all).
Jochmann et al. [90]	Measurement of FMD, before and 2 h after ingestion of either 500 mL water (control), black tea, or green tea in a cross-over study.	21 healthy postmenopausal women. Average age: 58.7 ± 4.5 years.	Randomized crossover study.	Green tea: from baseline of 5.4 ± 2.3% to 10.2 ± 3% 2 h, *p* < 0.001 Black tea: from baseline of 5 ± 2.6% to 9.1 ± 3.6% 2 h after black tea consumption; *p* < 0.001
Grassi et al. [91]	Five treatments with a twice daily intake of black tea (0, 100, 200, 400, and 800 mg tea flavonoids/day) in five periods lasting 1 week each.	19 healthy men ranging from 18 to 70 years.	Randomized crossover study.	Black tea dose dependently increased FMD from 7.8% (control) to 9.0, 9.1, 9.6, and 10.3% after the different flavonoid doses, respectively (*p* = 0.0001).
Suzuki-Sugihar et al. [92]	Two sessions in which green tea capsules containing 1 g of catechins or placebo capsules were taken. Test days were separated by at least a 2-week washout period.	19 healthy male volunteers ranging from 25 to 53 years.	Randomized crossover study	Green tea could reduce oxLDL in human participants. ↑ sTAC value 1 h after intake (*p* < 0.001).
Dower et al. [94]	(−)-epicatechin (100 mg/day), quercetin-3-glucoside (160 mg/day), or placebo capsules for a period of 4 weeks, in random order. 4-week washout periods.	37 healthy (pre)hypertensive men and women (40–80 years).	Double-blind placebo-controlled randomized clinical trial.	↓ sE-selectin by 27.4 ng/mL (*p* = 0.03) ↓ IL-1β by 20.23 pg/mL (*p* = 0.009) *Z* score for inflammation by 20.33 (*p* = 0.02)
Larson et al. [95]	Intake of a single-dose of purified quercetin aglycone (1095 mg) or placebo.	5 normotensive men (*n* = 5; 24 ± 3 years; 24 ± 4 kg/m^2^) and 12 stage 1 hypertensive men (41 ± 12 years; 29 ± 5 kg/m^2^).	Double-blind, placebo-controlled, crossover study.	↓ BP of stage 1 hypertensive men.
Kuntz et al. [99]	330 mL of beverage (placebo, juice and smoothie with 8.9, 983.7, and 840.9 mg/L of anthocyanin, respectively, for 14 days. 10-day washout periods	30 healthy female volunteers, age between 23 and 27 years.	Double-blind, placebo-controlled, crossover study.	Anthocyanin beverages: ↑ SOD, catalase, Trolox ↓ MDA
Davinelli et al. [100]	Intake of a standardized extract of maqui berry (162 mg anthocyanins) or a matched placebo, given 3 times daily for 4 weeks.	42 overweight volunteer smokers, aged between 45 to 65 years.	Double-blind, placebo-controlled design.	↓ oxLDL and 8-iso-prostaglandin F2α
Zhang et al. [101]	Intake of two anthocyanin capsules (320 mg anthocyanin/capsule) or placebo capsules twice daily for 24 weeks.	150 hypercholesterolemic individuals, age between 40 to 65 years.	Randomized, double-blind, placebo-controlled trial.	Anthocyanin group: ↓ CXCL7, CXCL8, CXCL12, CCL2. Positive association between CXCL7 and CCL2 with LDL-c, hsCRP and IL-1β. Negative correlation between CXCL8 and HDL-c. Positive correlation between CXCL8 and sP-Selectin.
Song et al. [102]	Consumption of four anthocyanins capsules/day (total of 320 mg/day) vs. placebo capsules for 24 weeks.	150 hypercholesterolaemic patients.	Randomized, double-blind clinical trial.	Anthocyanin group: ↓ β-TG, sP-selectin, and RANTES. Inhibition of pro-inflammatory and pro-thrombotic factors.
Hodis et al. [107]	Intake of daily doses of 25 g soy protein containing 91 mg aglycon isoflavone equivalents or placebo for 2.7 years.	350 postmenopausal American women, between 45 to 92 years of age, without diabetes and CVD.	Double-blind placebo-controlled randomized clinical trial.	CIMT progression in −16%.
Bhatt et al. [116]	Intervention group: 250 mg/Once Daily resveratrol capsule supplementation + oral hypoglycemic agents vs. control group: oral hypoglycemic agents for a period of 3 months.	62 patients with T2DM, aged between 30 and 70 years.	Prospective, open-label, randomized, controlled study	Resveratrol: ↓ hemoglobin A(1c), SBP, total cholesterol and LDL-c. No changes in HDL-c.
Movahed et al. [117]	Daily: 1000 mg of resveratrol capsule supplementation+oral hypoglycemic agents vs. 1000 mg of placebo capsule supplementation +oral hypoglycemic agents for a period of 45 days.	66 patients with T2DM, aged between 20 and 65 years.	Randomized placebo-controlled double-blinded parallel clinical trial.	Resveratrol: ↓ hemoglobin A(1c), glucose, insulin, insulin resistance, and SBP. ↑ HDL-c
Tomé-Carneiro et al. [118]	Intake of one capsule (350 mg) daily of GE-RES (8 mg resveratrol), GE or placebo for 6 months.	75 patients with T2DM, aged between 18 and 80 years.	Triple-blind, randomized, placebo-controlled trial	GE-RES: −20% of oxLDL (*p* < 0.001), −9.18% of ApoB (*p* = 0.014), −4.5% of LDL-c (*p* = 0.04). +8.5% non-HDLc (total atherogenic cholesterol load)/ApoB
Imamura et al. [119]	Intake of 100-mg resveratrol tablet or placebo tablet for 12 weeks.	50 eligible patients with T2DM (HbA1c > 7.0%). Average age 57–58 years.	Randomized, double-blind placebo-controlled clinical trial.	Resveratrol: ↓ SBP, CAVI, and d-ROMs
Agarwal et al. [120]	Intake of 400 mg trans-resveratrol, 400 mg grape skin extract, and 100 mg quercetin (RESV GROUP) or a cellulose placebo for 30 days	44 healthy subjects, >18 years.	Randomized, double-blind placebo-controlled clinical trial.	RESV GROUP: ↓ IL-8,IFN-γ, sVCAM-1, sICAM-1, and ↓ fasting insulin
**Olive oil** Camargo et al. [135]	MD+EVOO, SFA-rich diet, CHO-PUFA diet for 3 weeks.	20 healthy and elderly people. Mean age: 67.1 years.	Randomized crossover design study.	MD + EVOO: ↓ NF-κβ, MMP-9, TNF-α, and MCP-1 and ↑ IκBα expression
Hernáez et al. [139]	MD + EVOO (50 mL daily) or nuts (30 g daily) vs. a LFD. Dietary habits were assessed with 137-item FFQ and a 14-item questionnaire. LDL atherogenic traits (resistance against oxidation, size, composition, cytotoxicity) after 1 year of intervention.	210 men and women aged between 55 and 80 years at high cardiovascular risk.	Multicenter, randomized, parallel-group trial.	↑ LDL resistance against oxidation (+6.46%) and LDL particle size (+3.06%). ↓ the degree of LDL oxidative modifications (−36.3%). LDL particles became cholesterol-rich (+2.41%) and less cytotoxic (−13.4%) compared to LFD.
Castañer et al. [140]	25 mL olive oil with a LPC (2.7 mg/kg) or a high polyphenol content (HPC: 366 mg/kg) for 3 weeks separated by 2-week washout periods.	180 healthy European volunteers aged 20–60 years.	Randomized, crossover, controlled trial.	The intake of polyphenol-rich olive oil reduces LDL oxidation and gene expression related to atherosclerotic and inflammation processes in PBMCs (CD40, MCP-1, ICAM-1, etc.).
Widmer et al. [141]	Daily intake of 30 mL of EVOO or EGCG+EVOO for 4 months.	52 volunteers with early atherosclerosis and over 18 years.	Randomized, double-blind, trial.	Improved endothelial function in both groups. EVOO group: ↓ sICAM, white blood cells, monocytes, lymphocytes, and platelets.
